# Comprehensive transcriptomic profiling reveals complex molecular mechanisms in the regulation of style-length dimorphism in *Guettarda speciosa* (Rubiaceae), a species with “anomalous” distyly

**DOI:** 10.3389/fpls.2023.1116078

**Published:** 2023-03-16

**Authors:** Zhonglai Luo, Zhongtao Zhao, Yuanqing Xu, Miaomiao Shi, Tieyao Tu, Nancai Pei, Dianxiang Zhang

**Affiliations:** ^1^ School of Life Sciences and Medicine, Shandong University of Technology, Zibo, China; ^2^ Key Laboratory of Plant Resources Conservation and Sustainable Utilization, South China Botanical Garden, Chinese Academy of Sciences, Guangzhou, China; ^3^ Key Laboratory for Plant Diversity and Biogeography of East Asia, Kunming Institute of Botany, Chinese Academy of Sciences, Kunming, Yunnan, China; ^4^ Research Institute of Tropical Forestry, Chinese Academy of Forestry, Guangzhou, China

**Keywords:** “anomalous” distyly, brassinosteroid signaling, *Guettarda speciosa*, heterostyly, molecular regulation, genetic convergence

## Abstract

**Background:**

The evolution of heterostyly, a genetically controlled floral polymorphism, has been a hotspot of research since the 19th century. In recent years, studies on the molecular mechanism of distyly (the most common form of heterostyly) revealed an evolutionary convergence in genes for brassinosteroids (BR) degradation in different angiosperm groups. This floral polymorphism often exhibits considerable variability that some taxa have significant stylar dimorphism, but anther height differs less. This phenomenon has been termed “anomalous” distyly, which is usually regarded as a transitional stage in evolution. Compared to “typical” distyly, the genetic regulation of “anomalous” distyly is almost unknown, leaving a big gap in our understanding of this special floral adaptation strategy.

**Methods:**

Here we performed the first molecular-level study focusing on this floral polymorphism in *Guettarda speciosa* (Rubiaceae), a tropical tree with “anomalous” distyly. Comprehensive transcriptomic profiling was conducted to examine which genes and metabolic pathways were involved in the genetic control of style dimorphism and if they exhibit similar convergence with “typical” distylous species.

**Results:**

“Brassinosteroid homeostasis” and “plant hormone signal transduction” was the most significantly enriched GO term and KEGG pathway in the comparisons between L- and S-morph styles, respectively. Interestingly, homologs of all the reported S-locus genes either showed very similar expressions between L- and S-morph styles or no hits were found in *G. speciosa*. BKI1, a negative regulator of brassinosteroid signaling directly repressing *BRI1* signal transduction, was identified as a potential gene regulating style length, which significantly up-regulated in the styles of S-morph.

**Discussion:**

These findings supported the hypothesis that style length in *G. speciosa* was regulated through a BR-related signaling network in which BKI1 may be one key gene. Our data suggested, in species with “anomalous” distyly, style length was regulated by gene differential expressions, instead of the “hemizygous” *S*-locus genes in “typical” distylous flowers such as *Primula* and *Gelsemium*, representing an “intermediate” stage in the evolution of distyly. Genome-level analysis and functional studies in more species with “typical” and “anomalous” distyly would further decipher this “most complex marriage arrangement” in angiosperms and improve our knowledge of floral evolution.

## Introduction

Various floral traits in angiosperms evolved to promote out-crossing and prevent selfing, including herkogamy, dichogamy, mirror-image flowers, *etc.* Heterostyly, a special form of herkogamy, is characterized by the presence of two (distyly, most common) or three (tristyly) floral morphs within a species, with the reciprocal placement of stigma and anthers (Barrett et al., 2000). It has long been considered a key contributor to angiosperm diversification, as the flowers of one morph can produce seeds only when they are pollinated by other morphs but will reject pollen from the same morph (Lewis and Jones, 1992; Barrett and Shore, 2008; Naiki, 2012; Kappel et al., 2017).

Heterostyly is a genetically controlled floral polymorphism reported in at least 28 angiosperm families, which has intrigued scholars since the age of Darwin (Lloyd and Webb, 1992; Naiki, 2012). It has received considerable attention and served as an ideal model system in examining frequency-dependent selection, Mendelian inheritance, as well as the evolution of self-incompatibility (Bateson and Gregory, 1905; Fisher, 1941; Piper et al., 1986). Early studies mostly focused on the morphology, pollination biology, inheritance, and population genetics of heterostylous species, while barely touching the molecular mechanism regulating style development, the core component for elucidating this “most complex marriage arrangement” (Darwin, 1864; Barrett, 2019).

With the development of high-throughput NGS technologies, candidate genes related to style-length regulation are becoming revealed in the last decade (Kappel et al., 2017). The genetic architecture of the *S*-locus was first explored in primrose (*Primula*). *CYP734A50* controlling style length and *GLO* regulating anther height were identified (Huu et al., 2016; Li et al., 2016). *CYP734A50* encodes protein inactivating brassinosteroids, a phytohormone that promotes cell elongation. Its expression was only detected in the short-styled morph of primrose, while the loss or inactivation of the gene led to a long style, indicating that the *CYP734A50* locus is hemizygous in the S-morph haplotype (Huu et al., 2016; Zhao et al., 2019). Shore et al. (2019) identified *TsBAHD*, which has high homology to genes inactivating brassinosteroids, as the potential gene regulating style length in distylous *Turnera* (Passifloraceae). *TsBAHD* was expressed in the S-morph but was absent from the L-morph. Our recent studies in *Gelsemium elegans* (Gelsemiaceae) revealed that the style-length gene *GeCYP* and *CYP734A50* in *Primula* may have arisen from duplication of *CYP734A1* (Zhao et al., 2022). These findings proposed a genetic convergence of the style-length regulation genes as they were hemizygous with the function of inactivating BRs (Barrett, 2019; Zhao et al., 2022).

Besides the BRs-related genes, other candidates were also investigated in several studies, and some are linked to the *S*-locus (Manfield et al., 2005; Li et al., 2007), while others may be downstream components in the development of floral heteromorphy (McCubbin et al., 2006). The *S-ELF3* gene in *Fagopyrum* is probably a transcription factor (TF) with pleiotropic effects (Yasui et al., 2012). Zhao et al. (2019) illustrated in *Primula oreodoxa* that many genes were co-expressed with *CYP734A50* and showed a negative association with style length. In addition, TFs involved in phytohormone signaling pathways were identified as potential genes regulating style length in *P. oreodoxa*. These findings strongly suggest that the genetic control of style-length dimorphism was far more complicated than ever expected. It may not be solely determined by *S*-locus genes and BR-inactivation but rather more likely involves other genes and metabolic pathways and varied among angiosperm families or even genera. To date, the molecular regulatory networks behind distyly remain unexplored in most families (Henning et al., 2020).

Species in these studies (like *Primula*, *Turnera*, and *Gelsemium*) all have “typical” distyly, i.e., the reciprocity of stigma and anthers is precise between L- and S-morph flowers. Nonetheless, this complex floral adaptation exhibits considerable variability in nature. Some taxa have significant stylar dimorphism but the anther height differs less, displaying imprecise reciprocity, which has been termed “anomalous” distyly by Barrett and Richards (1990). It’s usually regarded as a transition in the evolution of distyly, although in some lineages, this stage may maintain for a considerable period (Charlesworth and Charlesworth 1979; Lloyd and Webb, 1992; Barrett et al., 2000). The genetic control of “anomalous” distyly, for example, in *Anchusa* and *Quinchamalium*, was suggested to be similar with that of typical distyly (Barrett and Richards, 1990). However, they frequently exhibited deviated morph ratios, either L-morph (*A. officinalis*, Philipp and Schou, 1981) or S-morph (*Q. chilense*, Riveros et al., 1987) individuals were in excess. These findings indicated that their floral syndrome and genetic structures differed from that of “typical” distylous species. However, the molecular basis for style-length regulation in these species is still unknown.

In the present study, we explored the molecular regulation mechanism of style-length dimorphism in *Guettarda speciosa* (Rubiaceae), a species with “anomalous” distyly. “Anomalous” distyly has also been reported before in the congeneric *G. scabra* (Barrett and Richards, 1990). Our previous research demonstrated it exhibited imprecise reciprocal herkogamy, and the reciprocity of stigma and anther heights is more precise at a higher level compared to a lower level (Xu et al., 2019). *G. speciosa* provides an ideal material to explore the regulation mechanism of style length in distyly evolution.

PacBio isoform sequencing (Iso-Seq) and organ-specific Illumina paired-end short reads sequencing were integrated to generate comprehensive full-length transcriptomes and to screen candidate differentially expressed genes. Long-read sequencing can complement short-read sequencing in transcripts quantification and gene identification, providing more reliable and accurate results. We aim at revealing the transcriptomic difference between L- and S-morph flowers and test the genetic convergence hypothesis. Specifically, we focus on: (1) Which genes and metabolic pathways may be responsible for the formation of “anomalous” distyly in *G. speciosa*? (2) Whether the mechanism differs from that of “typical” distylous species?

Answers to these questions would further elucidate the molecular basis of this particularly interesting breeding system in angiosperms and shed more light on the evolution of floral form and function.

## Materials and methods

### Study species and material preparation

**Figure 1:**
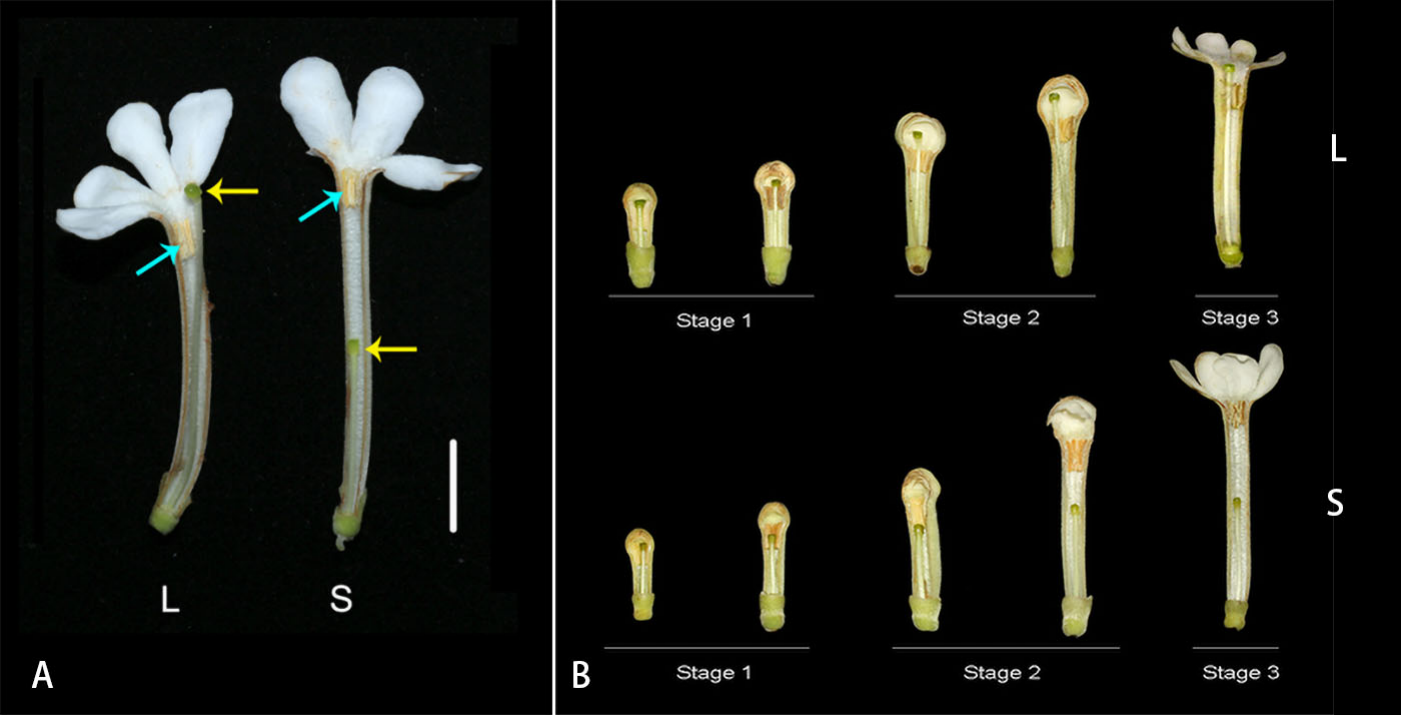
**(A)** Longitudinal dissection of *Guettarda speciosa* flowers, showing the positions of anther and stigma in L-morph (left) and S-morph (right). Yellow arrows indicate the position of stigmas, while blue arrows indicate the position of anthers. Bar = 10 mm. **(B)** The development process of L- and S-morph flowers, showing the three stages for RNA-seq material sampling.

*Guettarda speciosa* is a tree of Rubiaceae widely distributed in tropical islands and coastal zones around the Pacific Ocean (Watanabe and Sugawara, 2015). The species has white, tubular flowers pollinated by hawkmoths. L-morph flowers of *G. speciosa* have long styles and low-level anthers with the stigma positioned above the anthers and slightly exserted out of corolla, while the S-morph exhibits short styles and high-level anthers with the stigma positioned below the anthers (**Figure 1A**). In the study site, Yongxing Island, the ratios of L-morph and S-morph individuals met the expected 1:1 equilibrium (Xu et al., 2019).

To facilitate the sampling of RNA-sequencing materials, flower buds were dissected in the lab, and style length and anther position were measured by a digital caliper (573-S, Mitutoyo, Japan). The sampling method follows Shore et al. (2019) and Zhao et al. (2022). Three developmental stages were divided based on the elongation process of styles and corolla tubes as well as the relative position of stigma and anthers (see Results). Fresh style and androecium materials from the three stages were collected in each morph: (1) small floral buds (6-9 mm in corolla tube length; the stigma and anther were similar in height), (2) large floral buds (14-18mm in corolla tube length; the separation between stigma and anther were gradually increasing), (3) early flowering stage (22-30 mm in corolla tube length; the elongation of style slowed down) (**Figure 1B**). The stamens of *G. speciosa* are adnate to the corolla tube, and therefore the androecium and corolla tube were sampled as a whole. For each sample, materials were collected from one individual, and four biological replicates were prepared. In total, 48 samples were collected, including 24 style samples (12 L and 12 S) and 24 androecium-corolla tube samples (12 L and 12 S). Fresh materials were frozen in liquid nitrogen immediately after collection.

### RNA extraction and quality assessment

Total RNA was extracted with the HiPure Universal RNA Mini Kit (Magen, Guangzhou, China) following the manufacturer’s instructions, then stored at -80°C until use. RNA quality was assessed by Agilent Bioanalyzer 2100 system (Agilent Technologies, CA, USA) before sequencing. RNA samples with RIN (RNA Integrity Number) > 7.0 and OD 260/280 > 2.0 were used for PacBio library preparation, Illumina library construction, and sequencing.

### PacBio library construction, sequencing, and reads-error correction

RNAs from all 48 samples (including the three developmental stages of the style and androecium-corolla tube) were mixed in equal amounts. Total RNA (~5 μg) was reversely transcribed into cDNA through a SMARTer™ PCR cDNA Synthesis Kit.

PCR amplification was performed using the KAPA HiFi PCR Kits. Size selection of the PCR product was performed using the BluePippin Size Selection system (Sage Science, USA), and the fragments with a length of 0.5-6 kb were retained. SMRTbell™ template preparation was done according to the manufacturer’s instructions. Libraries were prepared for sequencing by annealing a sequencing primer in the SMRTbell Template Prep Kit, and binding polymerase to the primer-annealed template. The quality of the cDNA library was assessed using Agilent Bioanalyzer 2100. A total of 30 ng of the library for each SMRT cell was sequenced using polymerase 2.0 and chemistry on the PacBio Sequel 2 platform (Pacific Biosciences Inc., CA, USA) with 10 h of movie times by Biomarker Tech. (Beijing China).

### SMRT sequencing data processing and analysis

Data generated from SMRT sequencing were processed with the SMRT Analysis software (v2.3.0, Pacific Biosciences). Raw reads were error-corrected and processed into ROIs (reads of insert) by the ToFu pipeline (GitHub version, Pacific Biosciences of California, Inc., Melon Ark, CA, USA). Low-quality reads (with N removal ratio >10% and reads where the number of bases with a mass value of Q ≤ 10 accounted for more than 50% of the reads) and reads containing connectors were removed to get high-quality clean data. FLNC (full-length, nonchimeric) transcripts were then determined by searching for poly-A tail signals and the 5′ and 3′ cDNA primers in ROIs. The clustering algorithm ICE (iterative clustering for error correction) was employed to obtain consensus isoforms, and FL consensus sequences from ICE were polished using Quiver (Chin et al., 2013).

### Illumina library construction and transcriptome sequencing

RNA libraries were prepared by the NEBNext^®^ Ultra™ RNA Library Prep Kit for Illumina^®^ (NEB, USA) according to the manufacturer’s instructions. Sequencing was performed on an Illumina Hiseq 2000 platform and paired-end reads (raw reads) were generated with a length of 150 bp. Raw reads with adaptors or undetermined bases (poly-N) were removed and reads with low quality (containing more than 50% bases with Q-value ≤ 20) were also discarded. Clean reads data were deposited in the Genome Sequence Archive (GSA) database with accession number CRA009255 in the BIG Data Center.

### Gene functional annotation and enrichment analysis

Unigene sequences were aligned by blastx to protein databases NR (http://www.ncbi.nlm.nih.gov/), Swiss-Prot (http://www.expasy.ch/sprot), KEGG (http://www.genome.ad.jp/kegg/), and COG (http://www.ncbi.nlm.nih.gov/COG/) and aligned by blastn to nucleotide databases NT with an e-value < 1.0E-5. GO (Gene Ontology) annotation was performed with AmiGO (Carbon et al., 2009) in three ontology categories: Biological Process (BP); Cellular Components (CC); and Molecular Function (MF). KEGG annotation was performed using the KEGG Automatic Annotation Server (KAAS) (Moriya et al., 2007), providing additional functional information showing the pathways that the transcript isoforms involved.

GO enrichment analyses between the two flower morphs were performed using the GSEA (Gene Set Enrichment Analysis) software (v. 4.2.3, Subramanian et al., 2005). GSEA is a tool of the second-generation pathway analysis approach, which could produce more accurate and less biased results by making use of the entire expression data sets (Barry et al., 2005; Khatri et al., 2012). KOBAS (Xie et al., 2011) software was employed to test the statistical enrichment of DEGs in KEGG pathways. Transcription factor prediction was performed by iTAK (Zheng et al., 2016), and genes were assigned to different families. *Arabidopsis* TFs in Plant TFDB 3.0 (Jin et al., 2014) were used as the reference TF database.

### Screening and analysis of differentially expressed unigenes

Clean reads obtained from Illumina sequencing were mapped to the consensus isoforms using Bowtie2 (Langmead and Salzberg, 2012). RSEM program was employed to estimate the expression abundances of unigenes (Li and Dewey, 2011). FPKM (the number of fragments per kilobase of exon per million mapped fragments) was used to represent the relative expression levels of each transcript. Differential expression analysis was conducted in R with the DESeq2 package (ver. 1.26.0), and the statistical test results (*p*-values) were corrected for multiple testing with the Benjamini-Hochberg false discovery rate (FDR). Sequences were assigned as DEGs if the FDR ≤ 0.05 and FC (fold change) ≥ 2 (log_2_FC ≥ 1 or log_2_FC ≤ -1) between two transcriptomes.

Pairwise comparisons were performed between L- and S-morph at different developmental stages for both styles and filaments (corolla tubes) to identify potential transcripts involved in phenotypic differences. We also compared gene expression among different stages within each morph.

### Identifying homologs of *S-*locus genes and phytohormone-related genes in *G. speciosa*

Nucleotide sequences of all the reported candidate *S*-locus genes from *Primula, Turnera, Linum*, and *Fagopyrum* were used for the BLAST searches (BLAST+ 2.12.0) against the *G. speciosa* transcripts to find the closest homologs. Although the BLAST score is not the most robust for predicting orthology, it’s still an efficient way of assuming designations, at least on the gene family level (Henning et al., 2020). Sequences with the best match (highest score and smallest E-value) were selected for analysis. Their expression data were then compared between the L- and S-morph, and Q-PCR was performed to verify the expression levels of screened genes.

Brassinosteroids, auxin, and gibberellins play crucial roles in plant organ growth (McSteen and Zhao, 2008; Depuydt and Hardtke, 2011; Zheng et al., 2019). Important genes related to these phytohormone signaling pathways were selected from pieces of literature, and their homologs in *G. speciosa* were identified by BLAST searches to compare the expressions in the two morphs.

### Co-expression network and protein-protein interaction (PPI) analysis

To explore genes potentially correlated to style-length regulation, the WGCNA package (v. 1.68) in R was employed to perform the weighted gene co-expression network analysis. Co-expressed genes may have similar biological functions (Panteris et al., 2007). Genes were filtered by removing those with FPKM < 0.5. In all, 20,337 unigenes from 24 style samples (including biological replicates) were used to construct the co-expression network. Hierarchical clustering of unigenes was conducted according to the topological overlap matrix; the resulting gene dendrogram was used for module detection with the dynamic tree cut method (minModuleSize = 30). Modules with similar expression profiles were merged with a height cut of 0.25. Gene connectivity was quantified by edge weight, which was determined through topological overlap measure. Connectivity was defined as the sum of weights across all edges of a node. Module-trait correlation analyses were performed to detect the association between gene modules and style length.

Protein-protein interactions are essential for most biological processes in living cells. Integrating gene expression profiles with PPI has received increasing attention in studies. In order to investigate the functions of candidate DEGs from a deeper view, we constructed PPI networks with the aid of STRING (https://string-db.org/). STRING is a comprehensive protein-protein interaction database containing over 5,000 organisms and > 2,000 million interactions (Szklarczyk et al., 2019). PPI networks were generated by mapping the protein sequences of DEGs to the database, and single nodes/self-interactions of proteins in the networks were removed. Subnetworks of interested proteins were exported to Cytoscape (3.7.2) for visualization. Subcellular localization annotation of proteins in the PPI network was retrieved from the UniProt database (https://www.uniprot.org/). The localization information was then imported to Cytoscape and illustrated with the aid of the Cerebral (Cell Region-Based Rendering and Layout, http://www.pathogenomics.ca/cerebral/) plugin.

## Results

### Floral characteristics of *G. speciosa*

Populations of *G. speciosa* involved two floral morphs with different style lengths. The stigma in L-morph was positioned above the anthers and slightly exserted out of the corolla, while S-morph flowers had a shorter style with the stigma positioned below the anthers. Anthers of both morphs were arranged on the upper part of the corolla tube of mature flowers near the mouth but were not exserted (**Figure 1A**, see M & M for detail). The stigma height of fresh open flowers is 34.27 ± 2.80 mm (L-morph) and 18.58 ± 1.70 mm (S-morph), while anther height is 27.46 ± 3.12 mm (L-morph) and 35.04 ± 3.66 mm (S-morph) (mean ± SE; n=20 for L- and S-morph flowers, respectively). The difference in anther height (7.58 mm) between the two morphs is not as great as that of the style length (15.69 mm).

The developmental process of *G. speciosa* flowers is shown in **Figure 1B**. Three developmental stages were divided for the collection of RNA-seq materials (corolla tube length 6-9 mm, 14-18 mm, and 22-30 mm, respectively). Stigma and anthers were at similar levels in flower buds approx. 6-8 mm (L-morph) or 7-9 mm (S-morph) in length. Compared with the elevating of anthers, the style elongated faster in L-morph but slower in S-morph, and, consequently, the separation of stigma and anther increased with the growth of floral buds in both morphs (stages 2). In mature floral buds, the growth of style and floral tube slowed down, and no obvious elongation was observed after the anthesis.

### Full-Length transcriptome sequencing and bioinformatics pipeline

A total of 20.5 Gb of clean reads were generated after filtering with SMARTLink 4.0. Reads of inserts (ROI) were screened with full passes ≥3 and the accuracy of sequence was set at ≥0.9, with an average length between 1kb and 6kb.

221,872 circular consensus sequences (CCS) were obtained, including 184,073 full-length non-chimeric (FLNC) reads with an average length of ~2000 bp, which had the 5′ and 3′ barcoded primers and the poly (A) tail simultaneously; and 37,799 non-full-length (nFL) sequences which lacked the 5’ primer, 3’ primer, or poly (A) tail.

The RS IsoSeq module of the SMRT analysis (v2.3.0) was used to cluster the FLNC reads, and a total of 82,554 consensus isoforms were obtained through ICE clustering. FL consensus sequences from ICE were polished by Quiver, and 80,213 high-quality consensus transcript sequences were generated. CD-HIT (Li and Godzik, 2006) was employed to remove the redundant transcripts. In all, 46,758 non-redundant sequences were obtained and their completeness was evaluated by BUSCO (Simão et al., 2015). The length distribution of consensus isoforms is shown in **Figure S1A**.

### Illumina transcriptomic sequencing

Considering the higher depth and accuracy of Illumina sequencing, short reads generated through Illumina HiSeq 2500 platform were used to improve the quality of FL transcript and to determine the gene expression levels of different samples.

In total, 24 style samples and 24 androecium-corolla samples from L- and S-morphs were included for RNA sequencing. For each sample, 6 Gb clean data were obtained. In total, we got ~1,227 million paired-end reads with Q30 > 92.0.

### Functional annotation and classification

**Figure 2:**
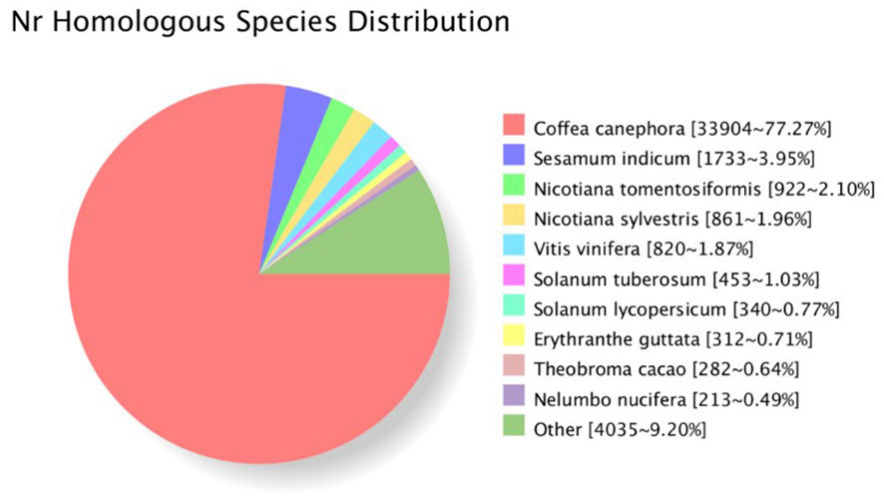
Species distribution of the top hits in homology search against the NR database.

A total of 44,370 open reading frames (ORFs) were identified. The distribution of the corresponding protein sequence lengths of the CDS is shown in **Figure S1B**. Protein sequences were used to search against eight databases (Nr, Swiss-Prot, COG, GO, KEGG, Pfam, KOG, eggnog) to annotate obtained unigenes. In total, 43,983 unigenes were successfully annotated in at least one database (with 43,882 in NR; 32,339 in Swiss-Prot; 35, 395 in GO; and 19,575 in KEGG). For *G. speciosa*, the top hit species was *Coffea canephora* (77.27%), followed by *Sesamum indicum*, and then *Nicotiana tomentosiformi*, suggesting it’s more closely related to coffee (**Figure 2**).

A total of 5354 GO terms in three ontology categories (Biological Process, BP; Molecular Function, MF; Cellular Components, CC) were assigned to 35,395 unigenes. Metabolic process (GO:0008152), Catalytic activity (GO:0003824), and Cell part (GO:0044464) are the most highly represented GO terms in BP, MF, and CC, respectively. Overall, 19,575 unigenes were matched to 128 KEGG pathways, with ‘biosynthesis of amino acids (ko01230, 724 genes),’ ‘carbon metabolism (ko01200, 716 genes)’, and ‘plant hormone signal transduction (ko04075, 582 genes)’ being the top three pathways with the most genes annotated. Many unigenes were also mapped to pathways, ‘biosynthesis of amino acids (ko01230)’, ‘starch and sucrose metabolism (ko00500)’, ‘amino sugar and nucleotide sugar metabolism (ko00520)’, and ‘ribosome biogenesis in eukaryotes (ko03008)’, suggesting active biosynthesis and metabolism in these tissues.

### Differential expression analysis of unigenes

Gene expression in styles and filament-corolla tubes between L- and S-morphs was compared respectively and analyzed to identify potential genes associated with the phenotypic differences during development. The number of DEGs differed between developmental stages. For the styles, there were 1381 (stage 1), 1643 (stage 2), and 1044 (stage 3) DEGs detected between the L- and S-morph, respectively. Many more genes were differentially expressed at the middle development stage, followed by the early stage, and the fewest genes showed differential expression at the late stage in intermorph comparisons (**Figure S3**). In all, 2914 genes were identified as DEGs which showed significant differential expression between L- and S-morph styles in at least one stage, and they were employed for further studies.

In the 2914 DEGs, 341 genes were identified as transcription factors (TFs, **Table S1**). The RLK-Pelle family TFs occupied the largest proportion (~30%, 100/341), including 44 RLK-Pelle_LRR members and 23 RLK-Pelle_RLCK members. Other important families include bHLH (14 unigenes), MADS (11 unigenes), AUX/IAA (9 unigenes), and BES1 (5 unigenes, **Figure S2**).

For the floral tube, there were 527 (stage 1), 824 (stage 2), and 435 (stage 3) DEGs detected between the L- and S-morph, respectively (FDR ≤ 0.05 and fold change ≥ 2). These were fewer compared with that of style tissues (**Figure S3**). In all, 1428 genes showed significant differential expressions between L- and S-morph in at least one stage.

### GO enrichment and KEGG pathway analysis

Gene Ontology (GO) enrichment analyses were performed with the aid of GSEA, which screens out gene sets with significant concordant differences between phenotypes, by making use of the entire expression data, instead of merely DEGs. It produces more accurate and less biased results for the enrichment tendency across different developmental stages.

**Figure 3:**
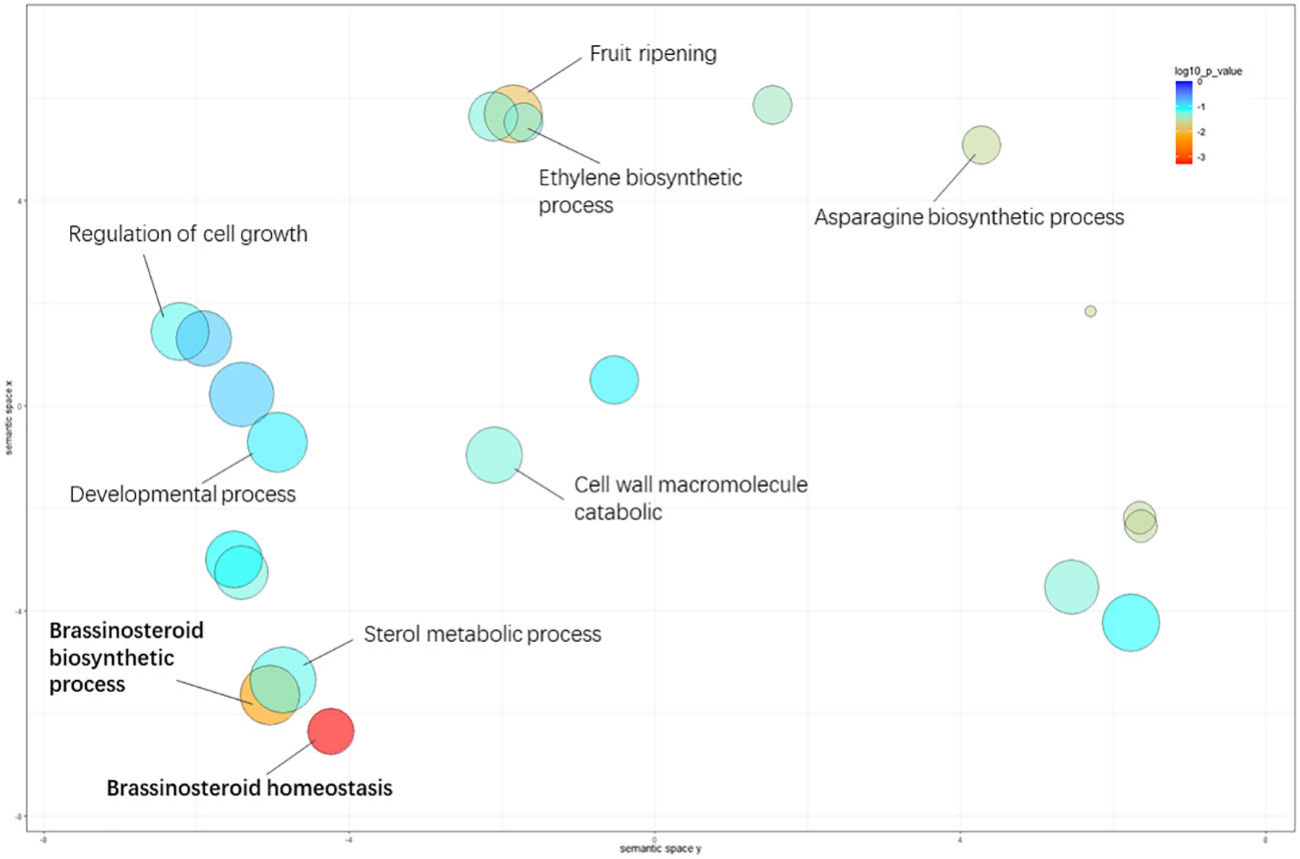
Significantly enriched GO terms in the comparison of L- vs. S-morph styles. The circle color represents the log10 transformed p-value in REVIGO analysis. The circle color represents the log10 transformed p-value in REVIGO analysis.

For biological processes, “brassinosteroid homeostasis” (GO: 0010268), “brassinosteroid biosynthetic process” (GO: 0016132), and “fruit ripening” (GO: 0009835) were significantly enriched between the L- and S-morph style, and “brassinosteroid homeostasis” was the most highly represented GO term, suggesting BR-mediated cellular activity involved in style elongation (**Table S2**.). The significantly enriched GO terms in GSEA analysis were illustrated in **Figure 3**.

**Figure 4:**
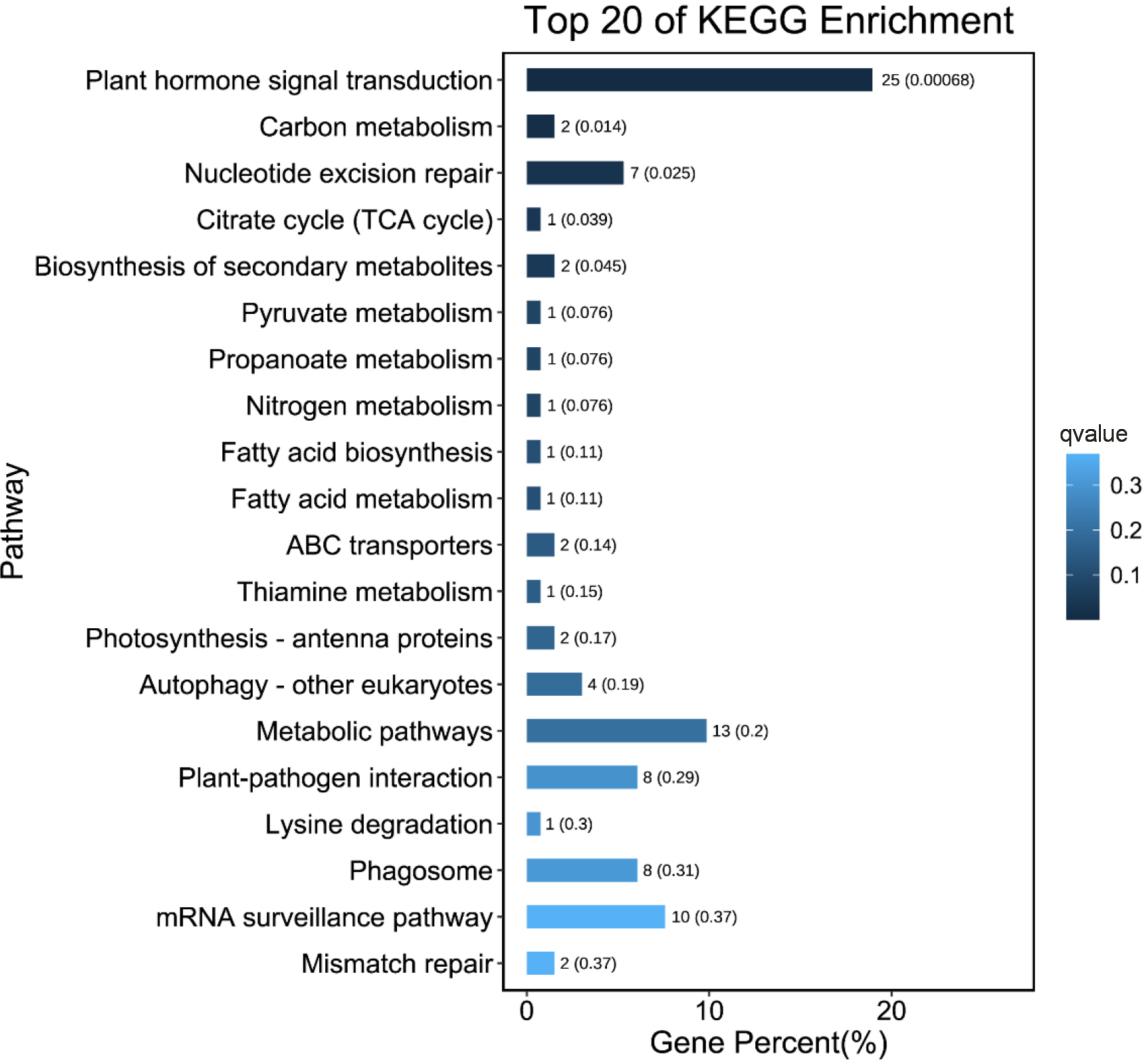
Top 20 enriched KEGG pathways for the DEGs between L- and S-morph styles. Smaller Q-value is shown in deeper blue.

In all, 128 pathways were predicted from the KEGG database. “Plant hormone signal transduction” (ko04075) was the most significantly enriched pathway in the comparisons between L- and S-morph styles (Q-value < 0.05) (**Table S2**). Most of the DEGs annotated to this pathway were brassinosteroid or auxin-related, including the *BKI1* (BRI1 kinase inhibitor 1, Gt_12638), *BZR1* (Brassinosteroid-resistant 1, Gt_67476), Auxin-induced protein *AUX22* (Gt_47484), Auxin-responsive protein *IAA7* (Gt_15651), and the Auxin influx carrier LAX2-like (Gt_6838). Interestingly, “plant hormone signal transduction” was the only significantly enriched pathway (Q-value < 0.05). The result of the KEGG enrichment analysis is summarized in **Figure 4**.

Between the floral tubes of L- and S-morph, there’s no significantly enriched GO term in GSEA analyses (**Table S3**). The only significantly enriched KEGG pathway was “photosynthesis - antenna proteins” (ko00196) (Q-value < 0.05, **Table S3**). Interestingly, no plant hormone-related GO terms or pathways were enriched between the L- and S-morph floral tubes.

### Identification of style length-related genes

To investigate the genetic basis of inter-morph style-length differentiation, potential DEGs were screened and analyzed coupling with the pathways that were involved. Brassinosteroids play essential roles in nearly all the processes of plant tissue growth and organ development with the important function of promoting organ elongation. To date, nearly all the relevant studies reported that the style-length regulation of distylous plants was dependent on BR-signaling pathways (e.g., Huu et al., 2016 in *Primula*; Shore et al., 2019 in *Turnera;* Zhao et al., 2022 in *Gelsemium*). Based on the functional enrichment analysis, we primarily focused on the expressional patterns of BR-related genes.

In the significantly enriched pathway, “plant hormone signal transduction” (k04075), DEGs *BKI1* (Gt_12638, **Table S2**), *BZR1* (Gt_67476), and *DWF4* (Gt_65179) were crucial genes in BR signaling. After examining their expression patterns and biological functions, *BKI1* (Gt_12638), which expressed significantly higher in S-morph styles across the three developmental stages (log2FC = -1.20, -1.13, -2.24 in stages 1, 2, 3, respectively. L- vs. S-morph; **Table S4**), was highlighted. *BKI1* was a negative regulator of brassinosteroid signaling, which directly repressed BRI1 signal transduction, and a higher *BKI1* level resulted in dwarf plants and reduced petiole (Wang and Chory, 2006; Jiang et al., 2015). Other DEGs not included in this pathway were also carefully examined, however, none of them seemed to have obvious relations with style-length differentiation.

To further elucidate the role of *BKI1* on brassinosteroid signaling, we examined the expression patterns of *DWF4* and *SAUR-AC1*, two crucial brassinosteroid-responsive genes. In S-morph styles, *DWF4* (Gt_65179) expressed higher while *SAUR-AC1*(Gt_53344) expressed lower compared to the L-morph styles (**Table S4**). This is in agreement with Wang and Chory (2006), who found that overexpression of *BKI1-YFP* led to a significant increase in the expression of *DWF4* and a decreased expression of *SAUR-AC1*in *Arabidopsis*.

### Transcriptional modules related to style length and PPI network analysis

Modules with co-expressed genes were detected by the R package WGCNA (v.1.69), and modules with similar expression profiles were merged through a height cut of 0.25 (75% similarity). In all, 22 modules were identified after merging, and module-trait analysis was performed to detect the association between modules and style length. The module “U” showed the most significant negative correlation with style length (cor = -0.70, p < 0.001). *BKI 1*(Gt_12638, Gt_35794) was included in this module. *DWF4* was found in the module “R”, which also negatively correlated with style length (cor = -0.51, p < 0.05; **Figure S4**).

In the module “J”, which had a significant positive correlation with style length (cor = 0.90, p < 0.001; **Figure S4**), the floral homeotic proteins *AG1* (Gt_15474), *AG2* (Gt_22594), and *AGL1* (Gt_31847) were found. Transcripts encoding expansin-A8 (Gt_21495, Gt_15039, and Gt_78377), an important protein required for rapid internodal elongation and causes loosening and extension of plant cell walls (Sampedro and Cosgrove, 2005), were also present in this module.

**Figure 5:**
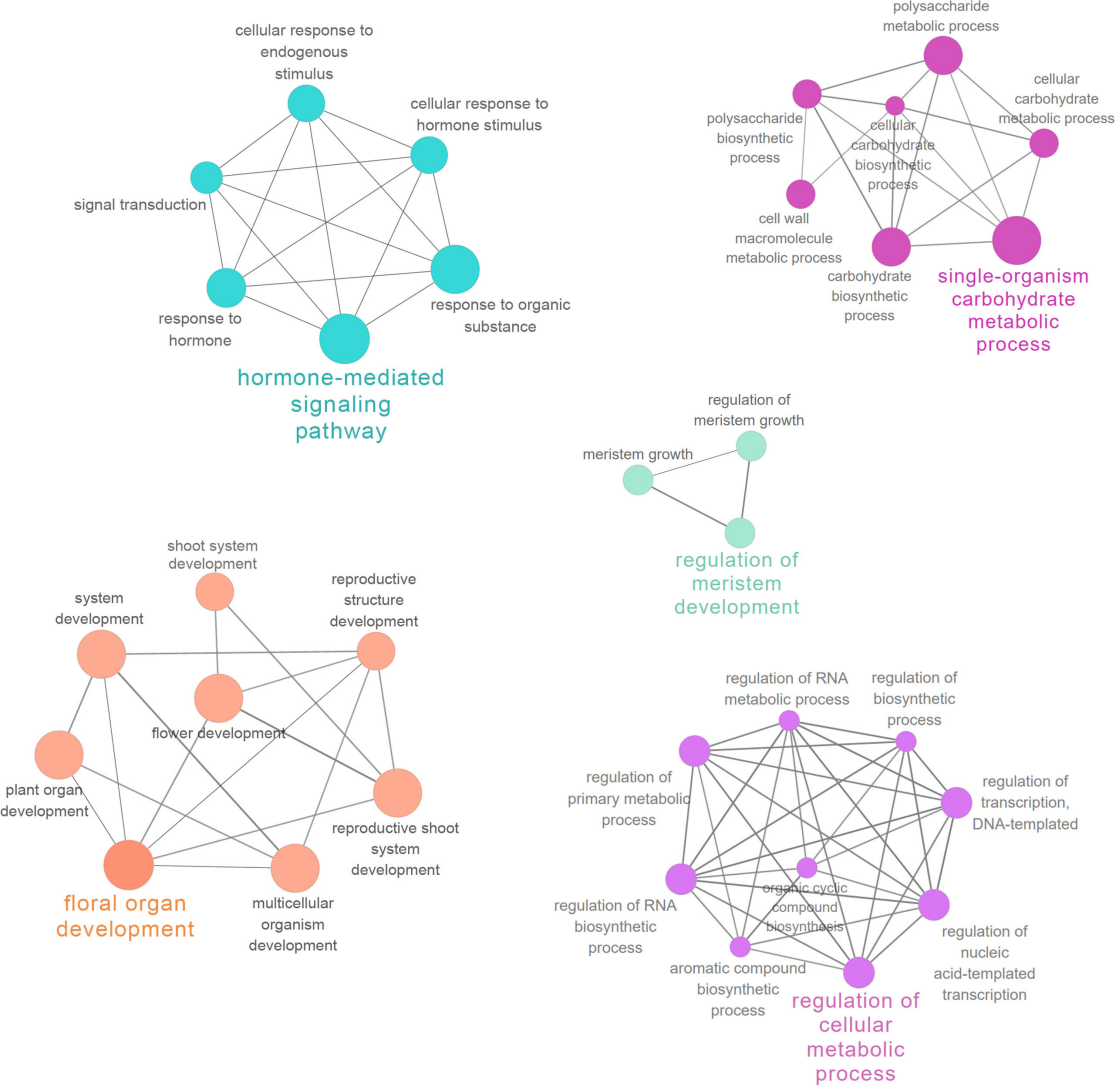
Functional enrichment analysis of PPI network. Important functionally grouped networks are shown in different colors.

PPI networks were constructed to illustrate the biological interactions of interested genes in greater detail. Firstly, protein sequences of 2914 DEGs in style were mapped to the STRING database and the generated network was exported to Cytoscape for further analysis. The PPI subnetwork was composed of 1163 nodes and 10976 edges (**Figure S5**). Functional enrichment analysis was performed with ClueGO (v.2.5.7) and CluePedia (1.5.7) (Bindea et al., 2009, Bindea et al., 2013). In the network, “hormone-mediated signaling pathway” (GO: 0009755), “floral organ development” (GO: 0048437), and “regulation of cellular metabolic process” (GO: 0031323) were all among the most significantly enriched GO terms (FDR < 0.001, **Table S5**). The functionally grouped networks are illustrated in **Figure 5**.

**Figure 6:**
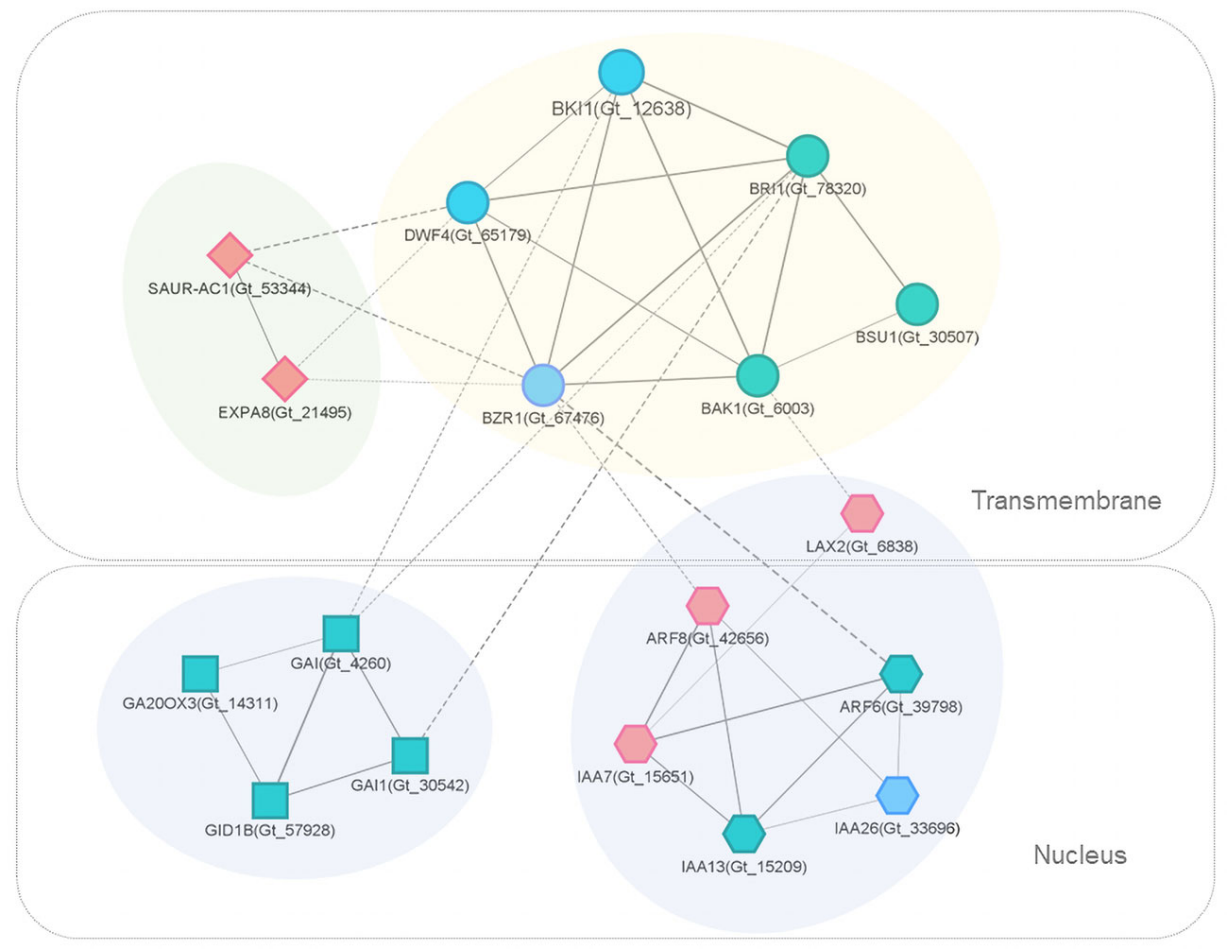
PPI subnetworks of key DEGs and related genes involved in BR (circular), auxin (hexagon), GA (square), and other important (diamond) signaling. Nodes represent proteins (genes), edges represent connections, and edge width represents the STRING interaction score. Solid and dash lines indicate within and between cluster interactions, respectively. For clear presentation, only edges with score ≥ 0.5 were presented. Different node colors indicate genes with significantly up-regulated (red), down-regulated (blue) or similar (grayish green) expression levels in the comparison of L-morph vs. S-morph styles (middle stage).

The interaction network of DEGs and related genes involved in brassinosteroid signaling was extracted for further examination (**Figure 6**). In L-morph styles, the expression of *BKI1* (Gt_12638) was significantly lower across the three developmental stages compared to S-morph. BKI1 interacted directly with several key proteins in BR-signal transduction, including BAK1, BZR1, DWF4, and BRI1; BKI1 showed strong connections (combined score ≥ 0.9) with them.

Interestingly, BR-subnetwork connected with several auxin-related proteins, including ARF6, ARF8, IAA7, IAA13, IAA26, and LAX2. *IAA7*(Gt_15651), *ARF8* (Gt_42656), and *LAX2* (Gt_6838) were all significantly up-regulated in L-morph compared with S-morph. LAX 2 is an auxin influx carrier of the LAX family involved in proton-driven auxin influx and facilitates acropetal auxin transport within tissues (Parry et al., 2001). While *IAA26* (Gt_33696), which acts as a repressor of early auxin response (Liscum and Reed, 2002), showed significantly lower expression in L-morph (**Table S4**), the gene encoding auxin-responsive protein IAA7 (Gt_15651) expressed higher in L-morph style. IAA7 was reported to promote growth in different types of organs (Liscum and Reed, 2002).

Another directly associated subnetwork consisted of several transcriptional regulators in the gibberellin (GA) signaling pathway, including the DELLA protein genes *GAI* (Gt_4260) and *GAI1* (Gt_30542), Gibberellin receptor gene *GID1B* (Gt_57928), and *GA20OX3* (Gt_14311). *GAI* acts as a repressor of GA responses (Peng et al., 1997). However, these transcripts expressed similarly in the L- and S-morph styles.

The subcellular locations of interested proteins were shown through the analysis of Cerebral (**Figure 6**). The brassinosteroids-related proteins, as well as SAUR-AC1 and EXPA8, were transmembranous. Most proteins in the auxin signaling-related subnetwork localized in the nucleus, while LAX2 was a transmembrane protein. All the DELLA proteins were located in the nucleus.

### The expression patterns of *S*-locus homologs and related genes in *G. speciosa*

**Figure 7:**
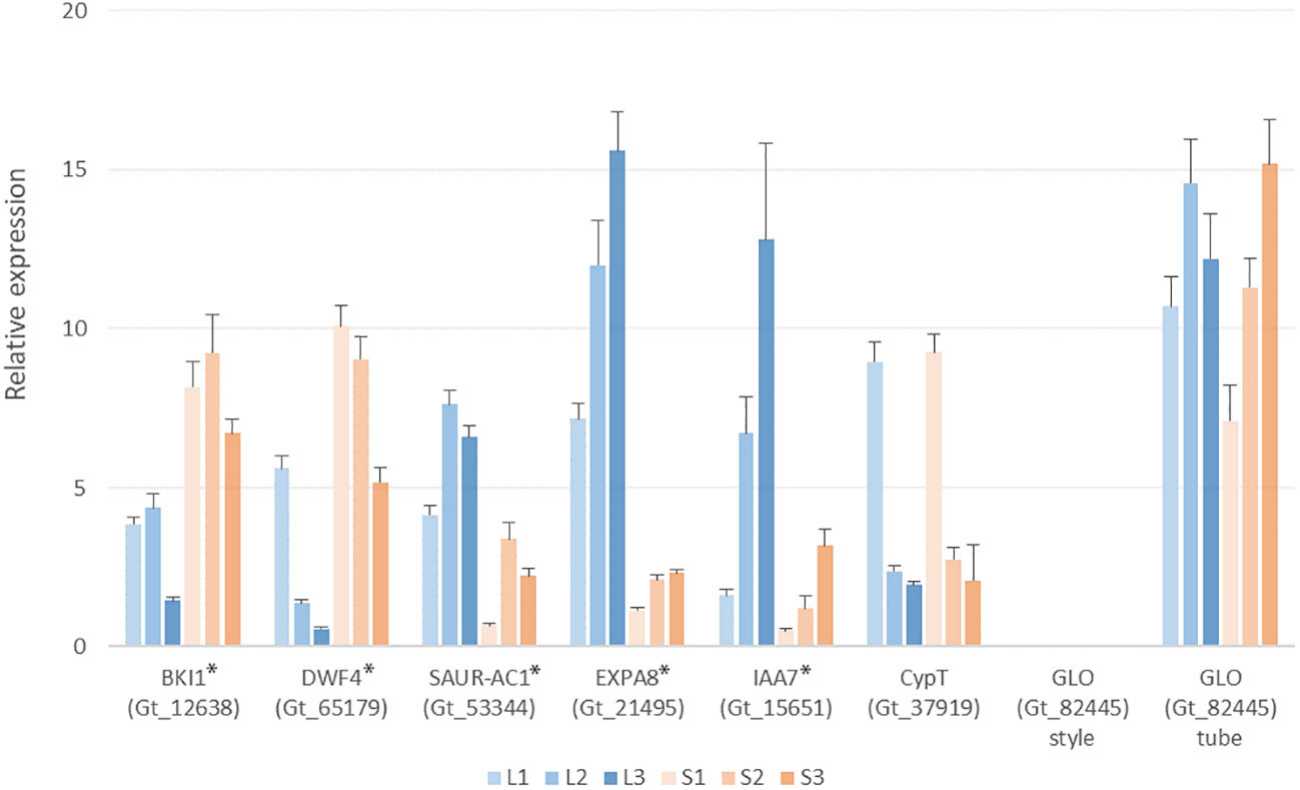
Q-PCR analysis showing expression levels of selected genes in the L- and S-morph styles of *Guettarda speciosa.* Error bars indicate the S.E. of three biological repeats. L1 (S1), L2 (S2) and L3 (S3) represents the three developmental stages, respectively. The tested genes showed significant differential expression between L- and S-morph (indicated by *), except *CypT* and *GLO*.

Since genes degrading brassinosteroids have been suggested responsible for style-length control in Primulaceae, Passifloraceae, and Gelsemiaceae, we focus on the brassinosteroid-related transcripts in *G. speciosa*. Based on functional annotation, several transcripts were matched with the keyword “brassinosteroid”. The gene encoding BKI1 (Gt_12638) expressed significant up-regulation in the S-morph style, but it also expressed in the L-morph style, indicating it’s not hemizygous. The expression patterns of *BKI1*, *DWF4*, and *SAUR-AC1* were verified by Q-PCR (**Figure 7**)

BLAST searches against the *G. speciosa* transcripts yield homologs of the distyly-related *S*-locus genes reported in other families. Homologs of *CYP734A50* (*CypT* in *Primula vulgaris*) (Gt_37919) and *CYP734A1*(*GeCYP* in *Gelsemium elegans*) (Gt_8662) expressed similarly between the L- and S-morph styles. For the other three S-morph-specific genes proposed in *Primula*, homologs of *PumT* (Gt_47787) and *KfbT* (Gt_82132) expressed similarly in both L- and S-morph, while no expression could be detected for the homolog of *GloT* (Gt_82445) (**Table S4**). Q-PCR results also validated that the expression of *CypT* (Gt_37919) was similar between the two morphs (**Figure 7**).

*TsBAHD* has been suggested to determine the short style in the S-morph of *Turnera*, while absent in the L-morph (Shore et al., 2019). Gt_9693 had high homology to *TsBAHD*, but it showed very similar expression patterns in both L- and S-morphs. Gt_78869 was homologous to *S-ELF3*, the suggested *S*-locus gene in *Fagopyrum*, but it’s not differentially expressed between the two morphs. *TSS1* is the S-morph-specific gene in *Linum*; however, there are no hits found in *Guettarda* (**Table S4**).

*GLO* has been reported to control the anther position in *Primula*, an S-morph specific gene that did not express in the L-morph (Li et al., 2016; Burrows and McCubbin 2017). Whereas, its homolog Gt_82445 shows very similar expressions in the floral tubes of both L- and S-morphs in *G. speciosa* (**Table S4**), which was consistent with the results of Q-PCR. The expressions of *EXPA8* and *IAA7*, two important DEGs in the phytohormone network, were also tested by Q-PCR (**Figure 7**).

## Discussion

The molecular regulation mechanism of style-length differentiation in distylous plants plays a central role in deciphering this unique breeding system as well as floral form evolution in angiosperms. Although evolutionary convergence was found in Primulaceae, Passifloraceae and Gelsemiaceae that hemizygous genes degrading brassinosteroids controlled style length, the mechanism for species with imprecise reciprocity, or "anomalous" distyly, was still unknown. Our results revealed that genes and pathways relating to brassinosteroids signaling instead of degradation may be involved in the style-length differentiation between the L- and S-morph flowers of *G. speciosa*. Moreover, transcriptional profiling and homologous search demonstrated that the candidate gene, *BKI1*, was not hemizygous, suggesting that the mechanism differed from the classic “*S*-locus” model.

### Transcriptional difference between the L- and S-morph flowers of *G. speciosa*

The style length of *G. speciosa* exhibited a significant difference between the two floral morphs. In mature flowers, L-morph styles were nearly twice as long as the S-morph styles (**Figure 1**; Xu et al., 2019). A number of genes were differentially expressed in style during the three developmental stages. Most DEGs were detected in the middle stage (stage 2), which was consistent with the development process that the length difference became more significant in this period (**Figure 1**). While in the late stage (stage 3), DEGs were fewer indicating that the differentiation was slowing down. This was in accordance with the findings in *Primula oreodoxa* where only a few DEGs were identified at the big floral bud stage (the late development stage) (Zhao et al., 2019).

GO analyses revealed that brassinosteroid-related biological processes were significantly enriched during style development. BR is a very important phytohormone playing dominant roles in organ growth and development patterning (Mussig et al., 2003; Yu et al., 2011; Wang et al., 2017), and “plant hormone signal transduction” (ko04075) was the most significantly enriched KEGG pathway. Several key genes in BR-signaling, such as *BKI1, DWF4*, and *BZR1*, were presented in this pathway. These findings strongly suggested that brassinosteroid was involved in the style length regulation of *G. speciosa.*

Fewer DEGs were detected in the floral tubes, and no plant hormone-related GO terms or pathways were enriched (**Figure S3**, **Table S3**), suggesting the insignificant transcriptomic difference between the L- and S-morph floral tubes. This is in agreement with the fact that the difference between anther heights is lesser than that of the styles. This further explains that the “anomalous” distyly in *G. speciosa* was mainly due to the similar positioning of anthers in L- and S-morph flowers.

### Expression patterns of the homologs of reported distyly-controlling genes

To date, the genetic regulation of style-length dimorphism has only been investigated in a very limited number of species from approximately six families. Serving as the most classical model in heterostyly studies, *Primula* has undoubtedly received extensive attention. *CYP734A50* involved in brassinosteroids degradation was the first identified “style-length gene” with experimental support, which has been reported in at least three *Primula* species (Huu et al., 2016; Li et al., 2016; Burrows and McCubbin, 2018; Zhao et al., 2019). In the most recent study in Gelsemiaceae, we revealed that the style length was also controlled by a gene of the *CYP734A* subfamily, *GeCYP* (Zhao et al., 2022).

A BAHD acyltransferase gene *TsBAHD* was proposed to regulate the style length in *Turnera* (Shore et al., 2019). Another two reported genes, *S-ELF3* in *Fagopyrum* and *TSS1* in *Linum*, were found to be short-morph specific, but their biological functions are still unknown (Ushijima et al., 2012; Yasui et al., 2012). In a study on the distylous aquatic plant *Nymphoides peltata* (Menyanthaceae), transcriptomes of the L- and S-morph flowers were compared, however, no style-length related genes were identified (Li et al., 2017).

Both *CYP734A50* and *TsBAHD* were suggested to inactivate brassinosteroids. Their homologs in *G. speciosa*, however, showed very similar expressions between the L- and S-morph styles, indicating that they were not possible to control style-length differentiation by regulating brassinosteroids and they were not hemizygous, either. Furthermore, no other genes for BR degradation were identified as DEGs. Homologs of *S-ELF3* are not differentially expressed between the two morphs, and no hits of *TSS1* are found in *Guettarda*. Expression data and network analysis also demonstrated that DEGs in the BR subnetwork were not hemizygous (**Table S4**). These findings suggest that the regulation mechanism of style length in “anomalous” distyly may be different from the reported cases depending on *S*-locus genes that degrade brassinosteroids.

The *S*-locus gene *GLO* was considered to determine anther position in the tubular *Primula* flowers, which is hemizygous and expressed in the S-morph rather than the L-morph floral tube. It did not express in the styles of both morphs, either. (Li et al., 2016; Burrows and McCubbin 2017; Zhao et al., 2019). *Guettarda speciosa* also have tubular flowers. But our data demonstrated that the *GLO* homologous gene (Gt_82445) expressed highly in the floral tubes of both morphs with very similar expression levels, and meanwhile, no expression was detected in styles (**Figure 7**). Therefore, *GLO* may not affect the anther height in *G. speciosa.* In the distylous *Turnera* with free filaments (filaments not fused with the floral tube), a plant-specific *S*-protein gene *TsSPH1* was suggested to likely determine the filament length, but the authors also admitted its precise roles were still uncertain (Henning et al., 2020). The molecular mechanisms controlling anther height may be different between species with free filaments and those having filaments adnate to the floral tubes. As the former may only involve filament elongation that depends on a “simple” difference of hormone regulation, while for the latter, anther height was affected by both the floral tube length and the position that anthers attached (e.g., *Primula* and *G. speciosa*). The anther position didn’t differ much in the L- and S-morph flowers of *G. speciosa*, which may explain why no relevant DEGs were identified through transcriptomic comparisons.

### Potential style-length regulation genes and networks in *G. speciosa*

Enrichment analyses and DEG screening suggested *BKI1*, the BRI1 kinase inhibitor 1 gene, to be an important candidate for style length regulation in *G. speciosa*. BKI1 is the only characterized inhibitor of transmembrane receptor kinases in plants, which acts as a negative regulator by limiting the interaction of BRI1 with its coreceptor, BAK1. *Arabidopsis* with *BKI1* inhibited by RNAi had significantly longer hypocotyls than the controls, and hypocotyl length was found to be negatively correlated with *BKI1* expression (Wang and Chory, 2006). Jiang et al. (2015) also observed enhanced BR signaling phenotype in the loss-of-function mutant *bki1-1* of *Arabidopsis*, including longer petioles and increased individual size. These findings demonstrated that *BKI1* plays a key role in the regulation of organ elongation through repressing brassinosteroid-related growth.

*BKI1* expressed significantly higher in the S-morph styles of *G. speciosa* across the three developmental stages and exhibited the highest expression in stage 2 (**Figure 7**.). This was consistent with the developmental process where the style of S-morph flower stopped elongation during the late second stage. In *Arabidopsis*, overexpression of *BKI1* resulted in dwarf plants with reduced stature and petiole length compared to controls (Wang and Chory, 2006), resembling the differentiated style length in S- and L-morph flowers of *G. speciosa*. Moreover, expression patterns of *DWF4* and *SAUR-AC1*, two genes used in *Arabidopsis* to test the influence of *BKI1* on BR signal transduction, also supported the fact that BKI1 acted as a negative regulator of brassinosteroid signaling. Interestingly, *BRI1*, one key gene in the BR signaling network, didn’t show differential expressions (**Figure 6**). This may be because BKI1 prevents the activation of BRI1 instead of suppressing *BRI1* expression (Wang et al., 2017).

It’s noteworthy that the BR-subnetwork connected with several proteins in auxin signaling. Positive regulators *IAA7*(Gt_15651)*, ARF8* (Gt_42656), and *LAX2* (Gt_6838) all significantly up-regulated, while *IAA26* (Gt_33696), a repressor of auxin response, down-regulated in L-morph style. It’s been demonstrated that the BR signaling pathway has extensive integration with many other phytohormone signaling pathways at both transcriptional regulation and signal transduction levels (Nemhauser et al., 2004; Wang et al., 2014). Auxin is known to act synergistically with BRs in promoting *Arabidopsis* hypocotyl elongation, through interactions among BZR1, ARF6, and genes regulating cell expansion (the expansins) (Lee et al., 2001; Goda et al., 2004; Oh et al., 2014). In *G. speciosa*, BKI1, BZR1, ARF6, ARF8, and Expansin-A8 interacted with each other (**Figure 6**). Zhao et al. (2019) reported that DEGs concerning BR, auxin, cytokinin, and gibberellin might potentially contribute to a style-length difference in *Primula oreodoxa.* Henning et al. (2020) also found that the *TsBAHD* gene intersected with additional hormone pathways such as the PIF network hubs which mediate red/far-red light signaling.

These findings suggested that style length in the Rubiaceous *G. speciosa* was primarily regulated by a BR-related signaling network, and *BKI1* may be the key gene that functions through differential expression. This differed from the reported cases with “typical” distyly (e.g., *Gelsemium* and *Primula*) that depending on the hemizygosity of the “*S*-locus” genes, may represent an “intermediate” stage in the evolution of distyly. We also found the BR subnetwork directly connected to the auxin signaling network, revealing the complexity in the molecular regulation mechanism of “anomalous” distyly. However, due to the lack of genomic data for *G. speciosa*, it’s infeasible to locate the candidate genes on chromosomes or conduct comprehensive evolutionary analyses in relevant genes. Functional studies on candidate genes and genomic analysis in more species with “typical” and “anomalous” distyly would further decipher this “most complex marriage arrangement” in angiosperms and improve our knowledge of floral evolution.

## Data availability statement

The datasets presented in this study can be found in online repositories. The names of the repository/repositories and accession number(s) can be found in the article/**Supplementary Material**.

## Author contributions

ZL, YX, and DZ designed the study. ZL performed most of the research and data analysis, and drafted the manuscript. ZZ performed some parts of the transcriptomic analysis. MS and TT participated in sample collection, and NP helped in data analysis. ZL, DZ, ZZ, and YX revised the manuscript. All authors contributed to the article and approved the submitted version.

## Supplementary material

Supplementary Figure 1 A. Read length distribution of consensus isoforms; B. Distribution of the protein sequence lengths coded by complete ORFs.

Supplementary Figure 2 The distribution of transcription factors in different gene families.

Supplementary Figure 3 Number of differentially expressed genes between L- and S-morph styles (blue) and floral tubes (orange) in three developmental stages.

Supplementary Figure 4 Module-trait relationships showing co-expressed gene modules correlated with the style length in WGCNA analysis. Each color cell contains the corresponding correlation and *p*-value (in brackets).

Supplementary Figure 5 PPI network constructed from the DEGs between L- and S-morph styles of G*uettarda speciosa*.
