# Supplementary figures and images for: Comprehensive transcriptomic profiling reveals complex molecular mechanisms in the regulation of style-length dimorphism in Guettarda speciosa (Rubiaceae), a species with “anomalous” distyly
Source: Front Plant Sci. 2023 Mar 16;14:1116078. doi: 10.3389/fpls.2023.1116078 (PMC10060554; doi:10.3389/fpls.2023.1116078)

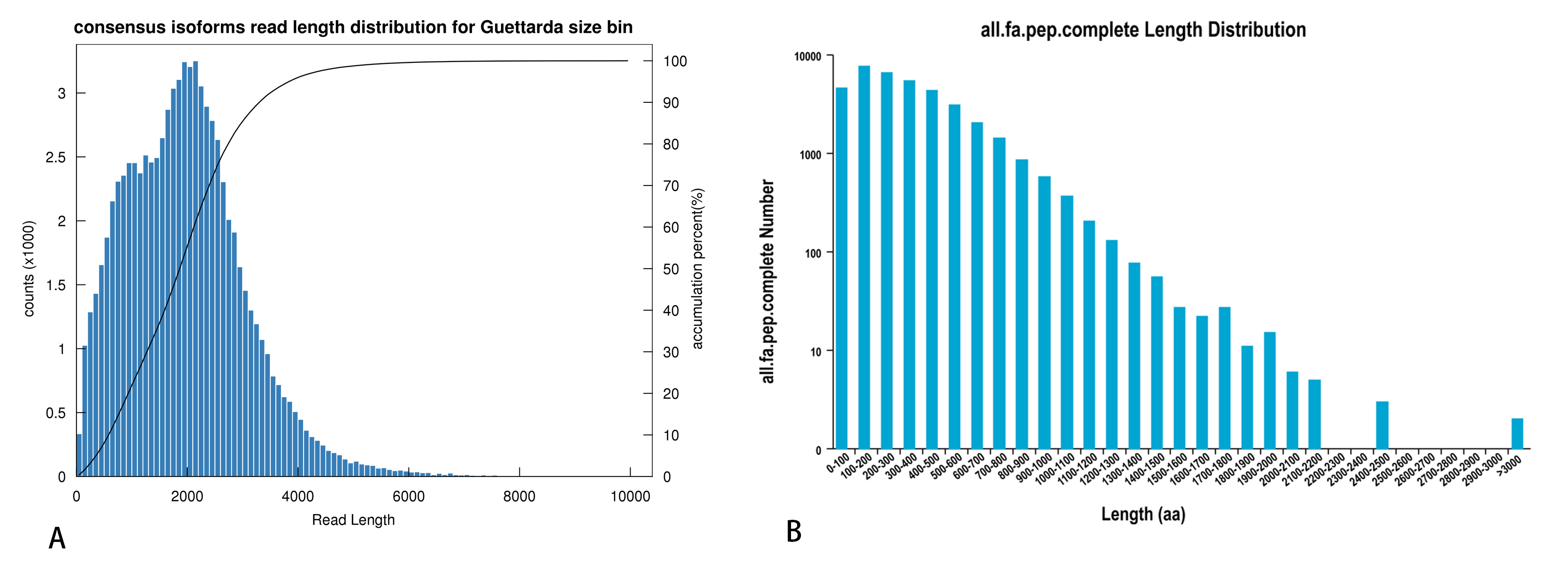

Supplement: Supplementary Figure 1 — A. Read length distribution of consensus isoforms; B. Distribution of the protein sequence lengths coded by complete ORFs. [file Image_1.tif]

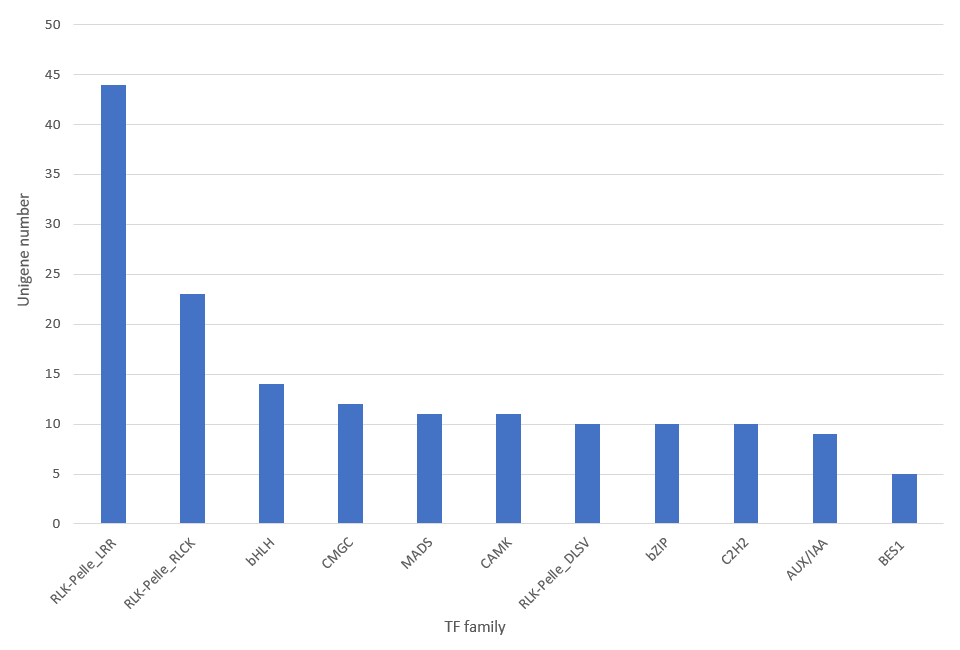

Supplement: Supplementary Figure 2 — The distribution of transcription factors in different gene families. [file Image_2.tif]

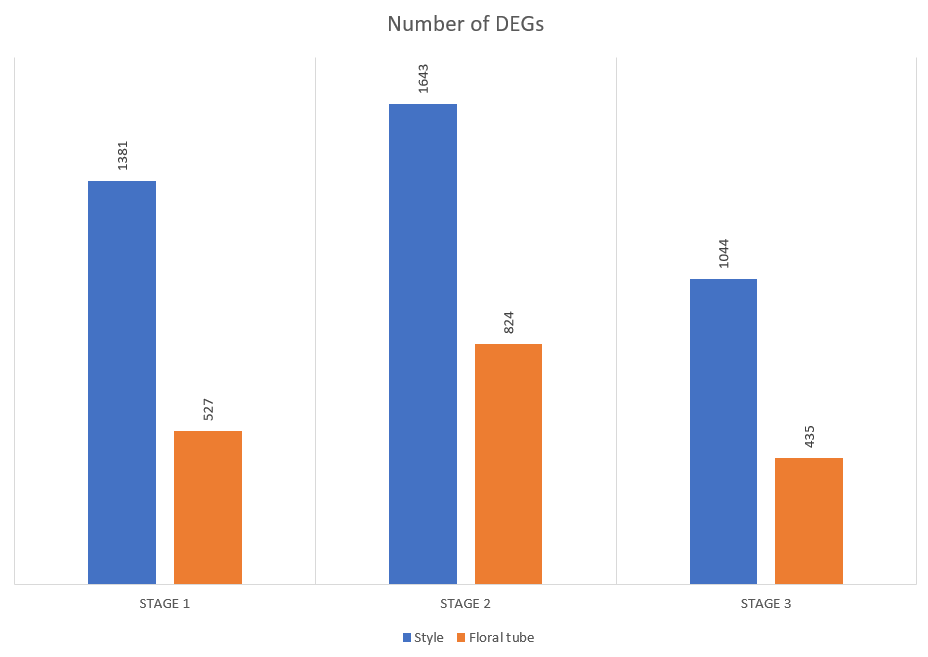

Supplement: Supplementary Figure 3 — Number of differentially expressed genes between L- and S-morph styles (blue) and floral tubes (orange) in three developmental stages. [file Image_3.png]

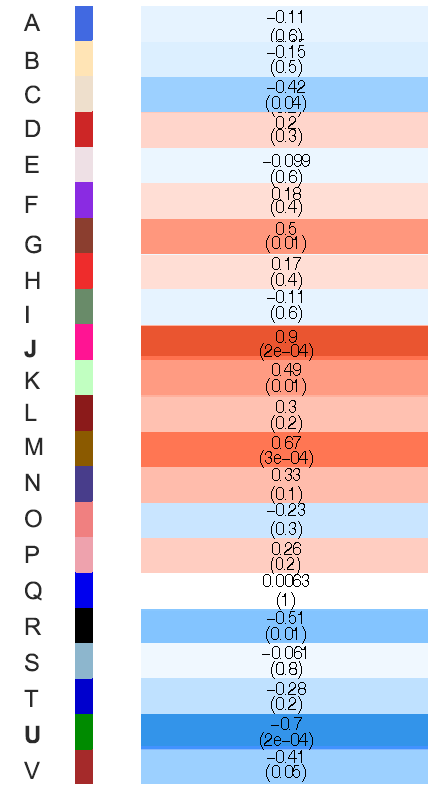

Supplement: Supplementary Figure 4 — Module-trait relationships showing co-expressed gene modules correlated with the style length in WGCNA analysis. Each color cell contains the corresponding correlation and p-value (in brackets). [file Image_4.tif]
